# Zinc Supplementation Improves Glucose Homeostasis in High Fat-Fed Mice by Enhancing Pancreatic β-Cell Function

**DOI:** 10.3390/nu9101150

**Published:** 2017-10-20

**Authors:** Vinícius Cooper-Capetini, Diogo Antonio Alves de Vasconcelos, Amanda Roque Martins, Sandro Massao Hirabara, José Donato, Angelo Rafael Carpinelli, Fernando Abdulkader

**Affiliations:** 1 Department of Physiology and Biophysics, Institute of Biomedical Sciences, University of São Paulo, São Paulo 05508-000, Brazil; viniciuscapetini@gmail.com (V.C.-C.); diogovasconcelos13@yahoo.com.br (D.A.A.d.V.); amandamartins@usp.br (A.R.M.); jdonato@icb.usp.br (J.D.); angelo@icb.usp.br (A.R.C.); 2 Institute of Physical Activity Sciences and Sports, Cruzeiro do Sul University, São Paulo 01506-000, Brazil; sandro.hirabara@gmail.com

**Keywords:** zinc, diabetes mellitus, insulin, insulin resistance

## Abstract

Zinc is an essential component of the insulin granule and it possibly modulates insulin secretion and signaling. Since insulin resistance is a hallmark in the development of type 2 diabetes mellitus, this study aimed at investigating if zinc supplementation is able to improve glucose tolerance and β-cell function in a model of insulin resistance. Male C57BL/6 mice were distributed in four groups according to the diet: normal fat (NF); normal fat supplemented with ZnCl_2_ (NFZ); high-fat (HF); and, high-fat chow supplemented with ZnCl_2_ (HFZ). Intraperitoneal glucose (ipGTT) and insulin (ipITT) tolerance, glycemia, insulinemia, HOMA-IR, and HOMA-β were determined after 15 weeks in each diet. Glucose-stimulated insulin secretion (GSIS) was investigated in isolated islets. The insulin effect on glucose uptake, metabolism, and signaling was investigated in soleus muscle. ZnCl_2_ did not affect body mass or insulin sensitivity as assessed by ipITT, HOMA-IR, muscle glucose metabolism, and Akt and GSK3-β phosphorylation. However, glucose tolerance, HOMA-β, and GSIS were significantly improved by ZnCl_2_ supplementation. Therefore, ZnCl_2_ supplementation improves glucose homeostasis in high fat-fed mice by a mechanism that enhances β-cell function, rather than whole-body or muscle insulin sensitivity.

## 1. Introduction

Diabetes mellitus is a growing public health problem affecting approximately 9% of the adult population worldwide [1]. While type 1 diabetes mellitus (T1DM) predominantly results from autoimmune destruction of pancreatic β-cells, with little or no insulin synthesis, type 2 diabetes mellitus (T2DM) develops due to the installation of insulin resistance in target tissues (reviewed in [2]), and high circulating insulin levels. This condition in the long-term can lead to a progressive exhaustion and ultimate destruction of the β-cells [3]. Currently, the treatment of T2DM is based on lifestyle modification, in addition to the administration of oral antihyperglycemic drugs or, in later stages, insulin treatment. However, an ancillary effect of zinc supplementation has been previously proposed to treat T2DM and its complications. Those studies have reported better glycemic control associated with the administration of this mineral [4,5,6], suggesting that zinc is a potential therapeutic agent for the treatment of diabetes. 

In the secretory granules of β-cells, insulin is associated with zinc in a stoichiometry of two zinc ions to six molecules of insulin. This combination stabilizes and prevents the degradation of insulin hexamers, protecting this hormone from the action of proteolytic enzymes [7]. In β-cells, the level of zinc appears to mediate various signaling pathways, suggesting that this mineral can act as a signaling molecule. In addition, zinc can also be a regulator of cellular metabolism [8], having antioxidant and anti-apoptotic effects. These effects occur via an enhancement in the cytosolic copper-zinc superoxide dismutase activity, metallothionein overexpression, or the inhibition of caspases and xanthine oxidase [9]. Additionally, zinc is an essential part of zinc finger-based transcription factors, such as transcription factor teashirt zinc finger homeobox 1 (Tshz1) and GLIS family zinc finger 3 (Glis3). The deficiency of these transcription factors is associated with the development of diabetes mellitus (DM) [10,11]. Therefore, an imbalance in the homeostatic control of zinc in these cells could cause significant damage in insulin secretion [12].

The effect of zinc on peripheral insulin sensitivity has also aroused the interest of many researchers. This mineral appears to be involved in insulin receptor signal transduction [13,14]. Zinc supplementation improves insulin sensitivity in adipocytes by activating hormone signaling pathways. Therefore, this mineral has insulin-mimetic effects [15,16], leading to the translocation of GLUT-4-containing vesicles to the plasma membrane [14]. Although it has been widely known that skeletal muscle is the predominant site of insulin-mediated glucose uptake [17,18] and that abnormalities in the insulin receptor signaling pathway in this tissue are associated with insulin resistance [19,20], there is little information about the metabolic effects of zinc on the insulin-signaling pathway in the skeletal muscle in DM.

While several studies have reported the participation of zinc in the synthesis and secretion of insulin, and on the control of insulinemia and glycemia [12,14,15], it is still unclear whether these positive effects on glycemic control in diabetic patients could be associated with an improvement in insulin secretion or sensitivity. The understanding of the molecular mechanisms of the action of zinc in DM would contribute to the development of targeted therapies and direct future research. In that regard, we have investigated in this study the effect of ZnCl_2_ in the control of insulin secretion and glycemia in a mouse model of glucose intolerance induced by high-fat chow. The results will be important to understand if the supplementation with ZnCl_2_ prevents or delays the manifestation of T2DM.

## 2. Materials and Methods

### 2.1. Animals, Treatments and Monitoring

After 11 days of weaning, male C57BL/6 mice were weighed and randomly divided into four experimental groups with the following dietary treatments for 15 weeks: (1) Normal-Fat chow (NF); (2) Normal-Fat chow, Zinc-supplemented (NFZ), in which the animals received normal-fat chow and supplementation with 20 mM ZnCl_2_ in water; (3) High-Fat chow (HF); and, (4) High-Fat chow, Zinc-supplemented (HFZ), that received high-fat chow and supplementation with 20 mM ZnCl_2_. The animals were maintained under controlled environmental conditions and had free access to chow and water (pH 5.0). The chow was adapted from the guidelines of the American Institute of Nutrition [21]. Pork lard was added in the high-fat chow in substitution to cornstarch. The normal-fat chow consisted of 69.9% carbohydrates, 10% fat, and 20.1% proteins. In contrast, the high-fat chow had 60% of its energetic value composed of fats, 19.9% carbohydrates, and 20.1% proteins. All of the procedures with animals were previously approved by the IACUC of the Institute of Biomedical Sciences, University of São Paulo, São Paulo, Brazil.

### 2.2. Glucose Tolerance and Insulin Sensitivity Tests

In the 14th week of treatment, animals were submitted to intraperitoneal glucose tolerance test (ipGTT) and insulin (ipITT). After 6 h of food restriction, animals had their glycemia measured by glucometer FreeStyle Lite^®^ (Abbott, Alameda, CA, USA), through blood sample collection from the tail, before (0 min) and 15, 30, 60, 90, and 120 min after an intraperitoneal administration of saline solution (0.9% NaCl) containing 20% glucose (at a dose of 1 mg glucose/g body weight). Then, the area under the curve (AUC) was calculated with the obtained values. The ipITT was performed 48 h after the ipGTT; after 4 h of food deprivation, animals had their glycemia measured using the same blood glucometer, before (0 min) and after 5, 10, 15, 20, 30, 40, and 60 min of the intraperitoneal administration of insulin Humulin^®^ (0.75 U/kg) (Lilly France, Fegersheim, FRA) [22]. The glucose decay constant rate (k_ITT_) was calculated as the slope of the linear regression of glucose concentration from 5 to 15 min after insulin administration [23].

### 2.3. Serum Measurements

After 15 weeks of treatment and 8h-fasting, animals were euthanized by cervical dislocation followed by decapitation. Serum samples were obtained for the measurement of glucose concentration using a glucose oxidase colorimetric kit (Liquiform^®^, Labtest, Lagoa Santa, MG, Brazil). Serum insulin concentration was determined by radioimmunoassay or by ELISA (Rat/Mouse Insulin Assay EZRMI-13K, Millipore Corporation, Billerica, MA, USA).

The tissue sensitivity to insulin and the functional capacity of pancreatic β-cell function to secrete insulin were assessed by homeostasis model assessment (HOMA). The fasting glycemia and insulinemia values were used to calculate the insulin sensitivity index (HOMA-IR) and the secretory capacity of the β-cell (HOMA-β), using the following equations: (a) HOMA−IR = glucose × insulin ÷ 22.5; and, (b) HOMA−β = 20 × insulin ÷ (glucose − 3.5), respectively [24].

### 2.4. Pancreatic Islet Isolation and Glucose-Stimulated Insulin Secretion (GSIS) Determination

Islets were isolated by digesting the pancreas with type IV collagenase solution (Sigma, St. Louis, MO, USA) (0.68 mg/mL) in Hanks’ buffer (in mM: NaCl 137, KCl 5, CaCl_2_ 1, MgSO_4_ 1, Na_2_HPO_4_ 0.3, KH_2_PO_4_ 0.4, NaHCO_3_ 4; pH 7.4) [25]. Islets were then hand-picked in Hanks’ buffer and groups of 5 islets, in triplicate, were pre-incubated in Krebs-Henseleit buffer (NaCl 115, CaCl_2_ 1, NaHCO_3_ 24, KCl 5, MgCl_2_ 1; pH 7.4 with 95% O_2_: 5% CO_2_), supplemented with 0.2% bovine serum albumin (BSA) *w*/*v* and 5.6 mM glucose, at 37 °C for 30 min. After pre-incubation, the solution was discarded and the islets were incubated for 1 h at 37 °C with the same buffer supplemented with 2.8 or 16.7 mM glucose. After glucose stimulation, the supernatant was retrieved and the total insulin content of the islets was extracted using an ethanol–water–HCl solution (52:17:1 *v*/*v*). Both supernatant and content samples were then frozen at −20 °C and subsequently assayed for insulin by radioimmunoassay.

### 2.5. Soleus Muscle Incubation and Protein Extraction

Soleus muscles of both limbs were quickly and carefully isolated after euthanasia, weighed, fixed in stainless steel staples maintaining the resting tension, and pre-incubated for 30 min, with O_2_/CO_2_ atmosphere (95%/5%), at 35 °C and 90 oscillations per min, in Krebs-Ringer-bicarbonate buffer (NaCl 118.5, NaHCO_3_, KCl 3.6, MgSO_4_ 0.5, CaCl_2_ 1.5, pH 7.4), containing 5.6 mM glucose and 1% BSA. Following the pre-incubation period, muscles were incubated for 20 min in the same conditions, in the presence or absence of 7 nmol/L insulin. Muscles were then homogenized in an appropriate buffer (Tris 100 mM, EDTA 1mM) supplemented with phosphatase (sodium pyrophosphate 10 mM; sodium fluoride 100 mM and sodium orthovanadate 10 mM) and protease inhibitors (PMSF 2 mM and Protease Inhibitor Cocktail^®^—Thermo Fisher, Chicago, IL, USA). After homogenization, 1% Triton X-100 was added to the homogenates and all samples were kept for 30 min at 4 °C prior to the centrifugation for 20 min at 340× *g* to remove all of the insoluble material. The Bradford method was used to determine the total protein content of the samples. The protein extracts were boiled for 5 min in a water bath and maintained at −80 °C for future analysis by Western blotting.

### 2.6. Western Blotting

Thirty micrograms of total proteins of soleus muscle were submitted to electrophoretic separation in 12% polyacrylamide gel containing sodium dodecylsulfate (SDS-PAGE 12%) in the Mini-Protean apparatus for minigel (BioRad, Hercules, CA, USA). Electrotransfer of proteins from the gels to nitrocellulose membranes was performed for 1 h at 15 V in a semi-dry apparatus (BioRad, Hercules, CA, USA). The membranes were incubated overnight at 4 °C, under agitation, in TSB (Tris 10 mM and NaCl 1.5 mM—pH 7.6) with 5% BSA to block nonspecific binding of antibodies to the membranes. Then, membranes were incubated overnight at 4 °C with primary antibodies against total Akt (#9272) and GSK3-β (#2710), phospho-Akt (Ser473) (#5473) and phospho-GSK3-β (Ser9) (#9336) (Cell Signaling Technology, Beverly, MA, USA). The antibodies were diluted (1:1000) in TSB with 3% BSA. Following that, membranes were incubated with a secondary HRP-conjugated antibody (Millipore, Temecula, CA, USA) for 60 min at room temperature and then incubated with a solution containing the chemiluminescence reagent (ECL) and exposed to photographic film. The intensity of the colored bands was determined by densitometry using Image Studio Digits 3.1^®^ software (LI-COR Biosciences, Lincoln, NE, USA). Band intensities were normalized to Ponceau staining to determine the relative protein expression [26].

### 2.7. Glucose Uptake and Metabolism in Isolated Soleus Muscle

Soleus muscles were isolated and pre-incubated, as described previously (Section 2.5). After pre-incubation, muscles were incubated for 1 h in the same conditions, in the presence or absence of 7 nmol/L insulin with the addition of 0.2 μCi/mL of 2-deoxi-[2,6-^3^H]-d-glucose, 0.3 μCi/mL of [U-^14^C]-d-glucose (Perkin Elmer, Waltham, MA, USA). A microtube containing 200 μL NaOH 2 M was added into the incubation flask for ^14^CO_2_ adsorption. After the incubation period, muscles were washed in cold PBS for one minute and then stored at −80 °C for further measurements. The medium used for the muscle incubation was acidified with 5 N HCl and held at 35 °C under constant stirring (180 oscillation per minute), for 1 h. The NaOH solution was then removed for the quantification of the ^14^CO_2_ released by muscle metabolism and absorbed by this solution. The muscles were digested in 300 μL of 1 M KOH, at 70 °C for 20 min, and 100 μL were reserved for the quantification of the uptake of 2-deoxy-[2,6-^3^H]-d-glucose. A saturated solution of Na_2_SO_4_ was added to the remainder for glycogen extraction in ethanolic solution, and then centrifuged at 4 °C for 15 min, at 425× *g*. The supernatant was discarded and the pellet resuspended and quantified for [U-^14^C]-glycogen synthesis and incorporation of [U-^14^C]-d-glucose into the muscle [27,28]. The radioactivity was quantified with Tri-Carb^®^ 2810TR counter (Perkin Elmer, Waltham, MA, USA) using a scintillation solution.

### 2.8. Statistical Analyses

Data (mean ± SEM) were analyzed with Graph Pad Prism6^®^ software (Graph Pad Software, Inc., La Jolla, CA, USA) by Two-way ANOVA following Bonferroni's multiple comparison post-test or by Student’s *t* test, when appropriate. A value of *p* ≤ 0.05 was considered for statistical differences. Only *p*-values less than 0.05 are reported in the figures.

## 3. Results

### 3.1. Body Mass and Intake of Diet, Water and ZnCl_2_

**Figure 1:**
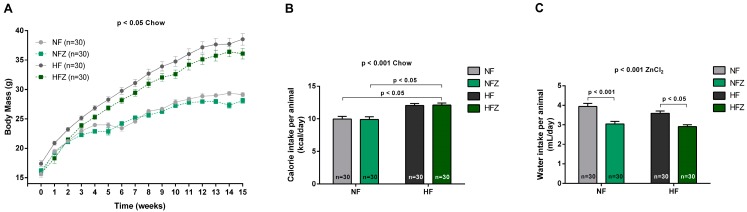
Body mass and caloric, hydric and ZnCl_2_ intakes. Body weight gain (**A**), calorie intake (**B**) and water intake (**C**) of animals of the NF and HF groups and those supplemented with ZnCl_2_. Values are means ± S.E.M. Two-way ANOVA: final body mass (chow, *p* < 0.01; zinc, *p* > 0.05; interaction, *p* > 0.05); calorie intake (chow, *p* < 0.001; zinc, *p* > 0.05; interaction, *p* > 0.05); and, water intake (chow, *p* > 0.05; zinc, *p* < 0.001; interaction, *p* > 0.05).

As expected, animals consuming high-fat chow gained more body mass in relation to those in normal-fat chow (*p* < 0.01) (Figure 1A). ZnCl_2_ supplementation did not interfere with the body weight gain. The higher body weight in animals fed the high-fat chow was associated with an increased caloric intake, when compared to normal-fat chow (*p* < 0.05) (Figure 1B). Throughout the treatment, ZnCl_2_-suplemented animals consumed less water than the NF (*p* < 0.01) and HF (*p* < 0.05—Figure 1C) groups. As expected, the ZnCl_2_ supplementation was reflected in increased Zn^2+^ daily intake in the supplemented groups, while no effect of chow was observed on Zn^2+^ intake (NF = 0.06 ± 0.0; HF = 0.09 ± 0.0; NFZ = 8.37 ± 0.3; and HFZ = 8.14 ± 0.2 mg Zn/day per animal — *n* = 30; 2-way ANOVA: chow *p* > 0.05, zinc supplementation *p* < 0.001, interaction *p* > 0.05; Bonferroni’s Post Test: NF vs. HF *p* > 0.05, NFZ vs. HFZ *p* > 0.05). 

### 3.2. Glycemia, Insulinemia, Homeostasis Model Assessment, Glucose Tolerance and Insulin Sensitivity

**Figure 2:**
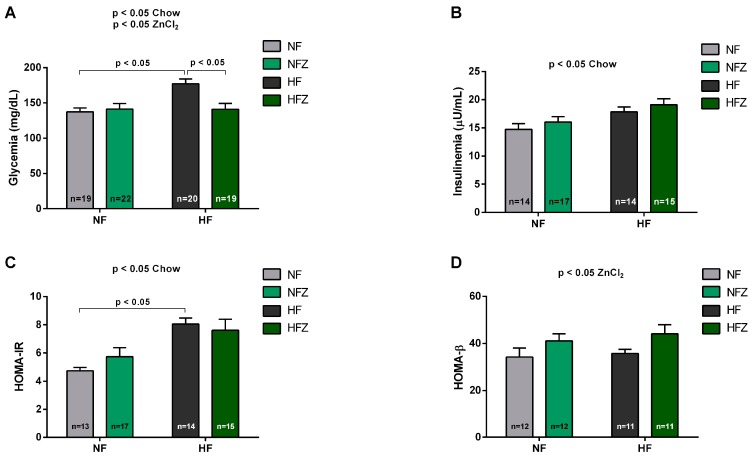

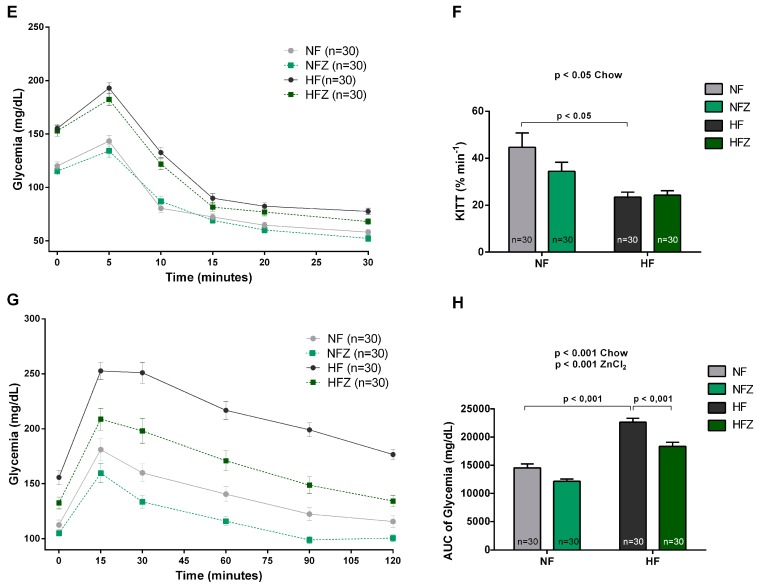
Serum parameters analysis. Glycemia (**A**), insulinemia (**B**), HOMA-IR (**C**), HOMA-β (**D**), ipITT (**E**), k_ITT_ ipGTT (**F**), ipGTT (**G**), and area under the curve (AUC) of ipGTT (**H**). Two-way ANOVA: glycemia (chow, *p* < 0.05; zinc, *p* < 0.05; interaction, *p* < 0.05); insulinemia (chow, *p* < 0.05; zinc, *p* > 0.05; interaction, *p* > 0.05): HOMA-IR (chow, *p* < 0.01; zinc, *p* > 0.05; interaction, *p* > 0.05); HOMA-β (chow, *p* > 0.05; zinc, *p* < 0.05; interaction, *p* > 0.05); k_ITT_ (chow, *p* < 0.01; zinc, *p* > 0.05; interaction, *p* > 0.05); and AUC ipGTT (chow, *p* < 0.01; zinc, *p* < 0.01; interaction, *p* > 0.05).

Fifteen weeks in high-fat chow led to hyperglycemia as compared to NF and HFZ groups (*p* < 0.05) and, notably, ZnCl_2_ supplementation prevented the increase in glycemia induced by high-fat chow (Figure 2A). Basal insulinemia showed no difference between the groups, although a significant effect of the chow was observed in the analysis (Figure 2B).

HOMA-IR and HOMA-β are indices that estimate, respectively, insulin resistance and β-cell secretory function [24]. The results of the HOMA-IR index indicated that high-fat fed groups were more insulin resistant as compared to the NF and NFZ groups (*p* < 0.05), and ZnCl_2_ supplementation was not able to prevent this dysfunction (Figure 2C). On the other hand, HOMA-β results suggested an improved secretory function of β-cells in ZnCl_2_-suplemented animals, regardless of the diet (*p* < 0.05) (Figure 2D). 

To study in more detail the insulin sensitivity in the experimental groups, an ipITT was performed (Figure 2E). In line with the HOMA-IR results, chronic consumption of high-fat chow significantly reduced the glucose decay constant (k_ITT_) (Figure 2F), and ZnCl_2_ supplementation produced no beneficial effects to prevent this insulin resistance. Then, an ipGTT was performed. In contrast to the ipITT results, ZnCl_2_ supplementation significantly improved the glucose tolerance either in mice consuming low- or high-fat diets. Furthermore, the area under the curve (AUC) (Figure 2G) of glycemia during the ipGTT (Figure 2H) was reduced in the HFZ group (*p* < 0.01), as compared to HF animals. Since the response to the ipGTT relies on endogenous insulin secretion, our findings collectively indicate that ZnCl_2_ supplementation improves glucose homeostasis via an increased glucose-stimulated insulin secretion.

### 3.3. Insulin Signaling and Glucose Metabolism in the Soleus Muscle

**Figure 3:**
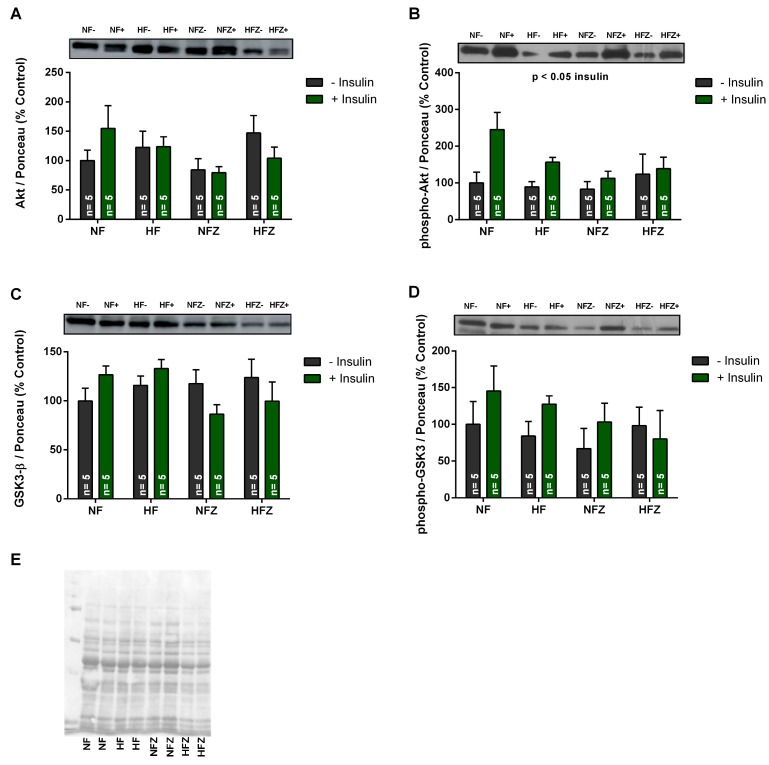
Effect of supplementation with ZnCl_2_ on the content and phosphorylation of Akt and GSK3-β in soleus muscle. Total Akt (**A**); phospho-Akt (Ser^473^) (**B**); total GSK3-β (**C**); phospho-GSK3- β (Ser^9^) (**D**); ponceau staining used for normalization (**E**). Two-way ANOVA: total Akt (chow, *p* > 0.05; insulin, *p* > 0.05; interaction *p* > 0.05); phospho-Akt (Ser^473^) (chow, *p* > 0.05; insulin, *p* < 0.05; interaction, *p* > 0.05); total GSK3-β (chow, *p* > 0.05; insulin, *p* < 0.05; interaction, *p* > 0.05); and, phospho-GSK3-β (Ser^9^) (chow, *p* > 0.05; insulin, *p* > 0.05; interaction, *p* > 0.05).

Previous studies have also described the changes in muscle insulin sensitivity after zinc supplementation [29,30]. Thus, despite no changes in whole-body insulin sensitivity in our zinc-supplemented animals, we further studied if zinc supplementation could affect insulin signaling and metabolism specifically in the skeletal muscle. The content of Akt and GSK3-β proteins in soleus muscle did not change among the groups. Insulin increased the phosphorylation of Akt, but not of phospho-GSK3-β, in the experimental groups. However, supplementation with ZnCl_2_ did not affect the response to insulin (Figure 3).

**Figure 4:**
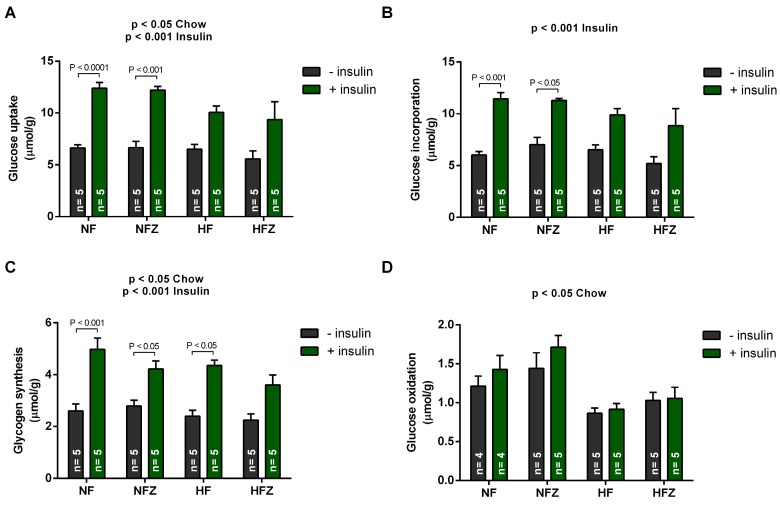
Uptake and metabolism of glucose in the soleus muscle. Glucose uptake (**A**), glucose incorporation (**B**), glycogen synthesis (**C**) and glucose oxidation (**D**) were assessed in the soleus muscle. Two-way ANOVA: glucose uptake (chow, *p* < 0.05; insulin, *p* < 0.01; interaction, *p* > 0.05); glucose incorporation (chow, *p* > 0.05; insulin, *p* < 0.01; interaction, *p* > 0.05); glycogen synthesis (chow, *p* < 0.05; insulin, *p* < 0.01; interaction, *p* > 0.05); and, glucose oxidation (insulin, *p* > 0.05; chow, *p* < 0.01; interaction, *p* > 0.05).

When glucose uptake and metabolism were evaluated in soleus muscle, we observed that insulin increased the uptake of 2-deoxy [2,6-3H]-d-glucose (Figure 4A), the incorporation of [U-14C]-d-glucose (Figure 4B) and glycogen synthesis (Figure 4C). On the other hand, high-fat chow reduced soleus glucose uptake, glycogen synthesis, and glucose oxidation (Figure 4). Importantly, zinc intake did not affect these responses, indicating no effect of supplementation directly on glucose metabolism in skeletal muscle.

### 3.4. Glucose-Stimulated Insulin Secretion (GSIS)

**Figure 5:**
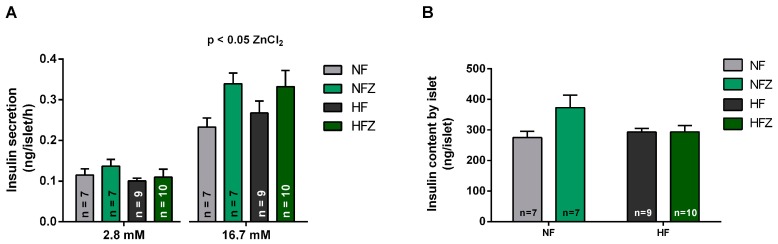
Glucose-stimulated insulin secretion (GSIS) and insulin content of isolated pancreatic islets. Effect of low (2.8 mM) and high (16.7 mM) glucose concentration in insulin secretion (ng/islet/h) (**A**). Islet insulin content (**B**). Two-way ANOVA of the insulin secretion by islets at 16.7 mM glucose (chow, *p* > 0.05; zinc, *p* < 0.05; interaction, *p* > 0.05).

To evaluate the effects of zinc supplementation now on β-cell secretory function, we performed incubations of freshly isolated islets from mice of the four experimental groups with different glucose concentrations. Consistent with our previous in vivo results, we observed an enhanced insulin secretion elicited by 16.7 mM glucose in the groups that received ZnCl_2_ supplementation (Figure 5A right). No effect of the treatments was detected at the low (2.8 mM) glucose concentration (Figure 5A left). The effects of zinc supplementation cannot be explained by changes in the insulin content in the islets, since no significant difference was detected in the islet insulin content among the groups (Figure 5B).

## 4. Discussion

**Figure 6:**
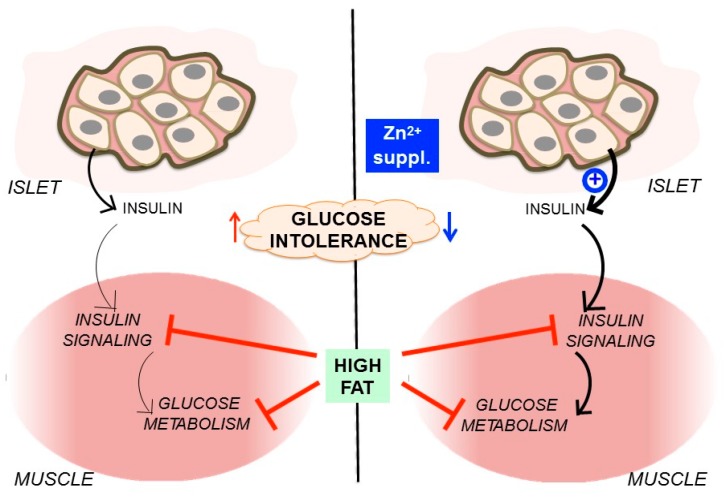
Improvement of glucose homeostasis by zinc supplementation in high fat-fed mice.

As expected, high-fat chow used in this study was able to generate an appropriate model of mouse obesity, insulin resistance, and glucose intolerance, which are typical characteristics of T2DM. Supplementation with ZnCl_2_ was efficient to improve glucose homeostasis in high fat-fed mice. The zinc effects were apparently mediated by a positive effect on β-cell function, improving insulin secretion, instead of affecting body mass, peripheral insulin sensitivity, or muscle glucose uptake, and metabolism (Figure 6).

Excessive intake of zinc for long periods may interfere with the metabolism of iron and copper, with consequences to the immune system and reducing HDL-cholesterol concentrations [31,32]. Although these biochemical markers were not investigated in the present study, previous studies reported no signs of toxicity or adverse effects in *ob/ob* mice supplemented with 20 mM ZnCl_2_ in the water for eight weeks [4]. The oral toxicity of ZnCl_2_ has been observed in rats, when the daily ingestion was administrated in excess (350 mg/kg) [33], but none of the groups supplemented in this study have reached this value. Additionally, zinc supplementation had no effect on body mass gain in the present study. These results are also another evidence of the lack of toxicity in the supplemented dose used. Furthermore, these results also suggest that ZnCl_2_ supplementation does not interfere with the endocrine and neural control of food intake, energy expenditure, and body mass. Despite it is possible that the ZnCl_2_ dissolved in water interfered with palatability, since a reduction in water intake was observed in animals receiving supplementation, we believe that this reduced water intake is most probably due to the increased electrolyte drinking in the supplemented animals, and thus a lower fluid intake to sustain whole body fluid and osmolyte balance. 

Chronic consumption of a fat rich diet is associated with hyperglycemia and insulin resistance [34,35]. Accordingly, the HF group became hyperglycemic, glucose intolerant, and insulin resistant. Despite some studies have suggested that zinc improves insulin resistance [13,14,36], the results of HOMA-IR, k_ITT_, muscle expression and phosphorylation of Akt and GSK3-β, and glucose uptake and metabolism in the soleus muscle showed that the ZnCl_2_ supplementation did not improve diet-induced insulin resistance. In fact, other authors have shown that supplementation with zinc can improve and/or mimic the action of insulin in adipose tissue [15,21], via an increase in glucose transport mediated by PI3-K and Akt [14] and the phosphorylation of GSK3-β [15,36]. However, the contribution of adipose tissue to the whole-body metabolism is limited, particularly when compared to skeletal muscle [37], which is quantitatively the major site of insulin-mediated glucose uptake [38]. Therefore, even if zinc supplementation could have improved glucose uptake in adipose tissue, this effect would possibly not be sufficient to reverse the whole-body insulin resistance induced by high-fat chow. In line with our results, Simon and Taylor [5] did not observe improvement in the tyrosine kinase activity of the insulin receptor in gastrocnemius muscle of *db/db* mice supplemented with 300 ppm of zinc for six weeks, confirming that the insulin resistance in skeletal muscle is not reversed or ameliorated by supplementation with this mineral.

Although insulin resistance was not improved, the supplementation with ZnCl_2_ increased insulin secretion in islets incubated with 16.7 mM glucose. HOMA-β results were also another evidence of increased efficiency of β-cells in ZnCl_2_-supplemented animals. The zinc effects on β-cell function were able to improve glucose homeostasis in high fat-fed mice. HFZ group showed a reduced hyperglycemia and a better glucose tolerance, as compared to HF group. Corroborating our results, Chen et al. [4] demonstrated that 20 mM ZnCl_2_ supplementation in water for eight weeks reduced the glycemia in *ob/ob* mice. Therefore, these results support the hypothesis that ZnCl_2_ optimized the functioning of the β-cells and consequently improved glycemic control. Also in line with our results, Nygaard et al. [12] showed that ZnCl_2_ is effective in improving insulin secretion and increasing the expression of zinc transporters ZnT3 and ZnT8 and MT1A in INS-1E cells. The effect in β-cells reported in the present study is apparently a general increase in β-cell function, not a protection against lesion, since zinc supplementation was associated with increased GSIS in both NFZ and HFZ groups, when compared to their respective non-supplemented groups.

Besides increasing GSIS, it is possible that zinc supplementation also decreases the availability of insulin to peripheral tissues, as zinc may increase the activity of the insulin-degrading enzyme (IDE) [39]. The IDE is the major enzyme responsible for insulin degradation in the liver and kidneys [40]. Thus, if IDE enzyme activity was increased in the groups supplemented with zinc, this could explain why we did not observe an effect on fasting insulinemia in these groups. However, zinc-supplemented animals exhibited an unaltered response to insulin infusion and an improved glucose tolerance, which suggest that any potential changes in the activity of IDE did not prevent the insulin effects on target tissues.

Among the possible mechanisms responsible for the β-cell-specific effect of zinc supplementation, one could consider changes promoted by zinc on the expression and/or the maintenance of proteins related to insulin processing in the β-cells, such as zinc transporters, MT, Cu-Zn SOD antioxidant enzyme, and ZnF, Tshz1, and Glis3 [10,14]. The study of the molecular mechanisms of zinc on the expression of these proteins may generate new knowledge about the mechanisms relating to the synthesis, storage, and, mainly, the secretion of insulin. The understanding of the influence of zinc on these mechanisms may allow the development of new therapeutic targets for the treatment of T2DM.

## 5. Conclusions

The results obtained in this study indicate an improvement in β-cell function and, consequently, in the glycemic control in animals supplemented with ZnCl_2_. Although the mechanism behind the improvement in β-cells function has not been identified, our findings suggest that supplementation with ZnCl_2_ can delay the manifestation of T2DM in a mouse model of diet-induced obesity.
